# Effects of grain-based pecking blocks on productivity and welfare indicators in commercial broiler chickens

**DOI:** 10.5713/ab.23.0384

**Published:** 2023-12-29

**Authors:** Byung-Yeon Kwon, Hyun-Gwan Lee, Yong-Sung Jeon, Ju-Yong Song, Jina Park, Sang-Ho Kim, Dong-Wook Kim, Kyung-Woo Lee

**Affiliations:** 1 Department of Animal Science and Technology, Konkuk University, Seoul 05029, Korea; 2 K-AniWel, Suwon 16672, Korea; 3 Department of Livestock, Korea National University of Agriculture and Fisheries, Jeonju 54874, Korea

**Keywords:** Animal Welfare, Broiler, Enrichment, Gut Health, Pecking Block

## Abstract

**Objective:**

This experiment was conducted to investigate the effect of grain-based pecking blocks on productivity and welfare status at two commercial broiler welfare-certified farms.

**Methods:**

Production and welfare indicators were assessed at two farms (designated Farm A and B). Both farms had two windowless houses with forced tunnel-type ventilation and housed broilers at stocking densities of approximately 16.7 birds/m^2^ (Farm A) and 16.8 birds/m^2^ (Farm B). Each house was divided into two or three equal sections and was provided with or without pecking blocks. Grain-based pecking blocks, measuring 25 × 25 × 25 cm, were given to broilers in both farms at 1 block per 1,000 birds. Various parameters including productivity (body weight and flock uniformity), corticosterone levels (in fecal droppings and feathers), footpad dermatitis, hock burn, feather dirtiness, gait score, litter quality, body surface temperature, and volatile fatty acids in fecal samples were assessed at 26 days of age, whereas litter quality was analyzed at 13 and 26 days of age.

**Results:**

There were no significant effects of providing pecking blocks on productivity (body weight and uniformity), fecal and feather corticosterone, welfare indicators (i.e., footpad dermatitis, hock burn, feather cleanliness, and gait score), and litter quality (i.e., moisture, nitrogen, and pH). No differences in body surface temperature between the control and enrichment treatments were noted in Farm B, but body surface temperatures of the head (p = 0.029) and legs (p = 0.011) in the enrichment vs. control group were elevated in Farm A. Butyrate concentration in the enrichment vs control group was higher in Farm B (p = 0.023), but this effect was not detected in Farm A.

**Conclusion:**

It is concluded that grain-based pecking blocks did not affect performance and welfare indicators. Further studies are warranted to elucidate the potential impact of grain-based pecking blocks on gut health indicators.

## INTRODUCTION

Commercial broiler industry has focused on the growth rate and feed efficiency since the mid-20th century with the advancements in breeding and rearing technologies [1]. Indeed, relative broiler growth exceeded more than 400% due to the selection pressure during 1957 to 2005. However, the unintended changes such as immune responses, musculoskeletal problems, and animal welfare issues have been emerged [1]. Welfare concerns in fast-growing broilers, led by high stocking density and litter moisture, can compromise the health of birds and induce restricted expression of natural behaviors [2]. To address these broiler welfare problems, it has become necessary to provide environmental enrichment that can stimulate birds’ natural behavioral expression [3].

Environmental enrichment refers to various forms of stimuli provided to the brain by the surrounding environment [4]. In other words, environmental enrichment can change the environment of animals to increase the possibility of expressing natural behaviors and to improve the biological functions of animals [5,6]. The examples of environmental enrichment used for broilers include perches, platforms, and pecking objects within the rearing environment [7]. Among the various behaviors exhibited by broilers, pecking behavior is an instinctive behavior specific to the species, and failure to satisfy this behavior could lead to stress and problematic behaviors [8]. Therefore, providing pecking objects is of utmost importance to fulfill these instinctive behaviors and to enhance animal welfare. Although pecking objects including mineral-based stones, hay bales, hanging strings, and laser lights have been used for broilers and laying hens, few experiments have been performed with the grain-based blocks which led us to set up the current experiment. We employed two animal welfare-accredited broiler farms to evaluate the production and physiological responses of commercial broilers provided with or without grain-based pecking block. Thus, this study was conducted to investigate the effects of the grain-based pecking blocks as an enrichment materials on productivity, animal welfare, and physiological indicators of broilers raised in animal welfare-certified broiler farms. The present study would increase our understanding the role of the pecking blocks in welfare indicators for broilers at the farm levels.

## MATERIALS AND METHODS

### Animal care

All experimental protocols and the use of broiler in the trial were approved by the Institutional Animal Care and Use Committee of Konkuk University (KUIACUC: KU21208-1).

### Farm selection

Experiment was conducted at two animal welfare-certified farms (Table 1). The first farm (designated as Farm A) was located in Boseong-gun, Jeollanam-do, and had 2 houses. Each house was 120 m long × 16 m wide (an area of 1,920 m^2^) and raised about 32,000 birds per house (the stocking density is 16.7 birds/m^2^). Fresh rice hulls were used as a bedding material. The second farm (designated as Farm B) was located in Yeongam-gun, Jeollanam-do, and and had 2 houses. Each house was 100 m long × 16 m wide (an area of 1,600 m^2^), and housed about 26,800 birds per house (the stocking density is 16.8 birds/m^2^). Contrary to farm A, recycled rice hulls were used in Farm B.

The house type of both farms was windowless with a forced ventilation of tunnel type. The broiler strain used was unsexed Arbor Acres provided from same integration company. Water and feed were provided *ad libitum*. Equal commercial starter and finisher diets were used for two farms. Two farms had 2 m-long wooden perches per 1,000 birds per house. The photoperiod was 23 hours light (L):1 hour dark (D) on day 0, and the daytime length gradually decreased until it reached 18L:6D on day 5. After that, it remained constant at 18L:6D until day 28. Other specifications were managed according to the recommended management manual suggested by the integrated company.

### Pecking blocks

**Figure 1:**
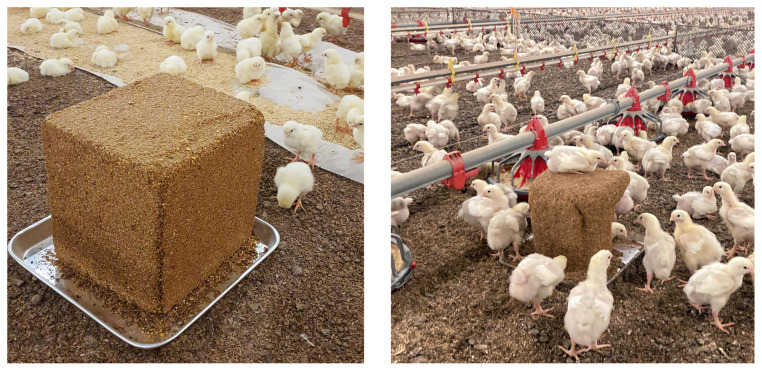
Grain block used in the experiment for enrichment. Block sized 25 × 25 × 25 cm, mixed with a grain base in the form of a cube. The block consisted of 50% to 60% of by-products as brans, 10% to 20% of grains, 10% to 15% of limestone, and other ingredients (moisture, molasses, and glycerin), and it was manufactured by mixing raw materials and applying pressure in a mold. One pecking block per 1,000 birds were supplied and all blocks supplied were completely consumed during the experimental period.

**Figure 2:**
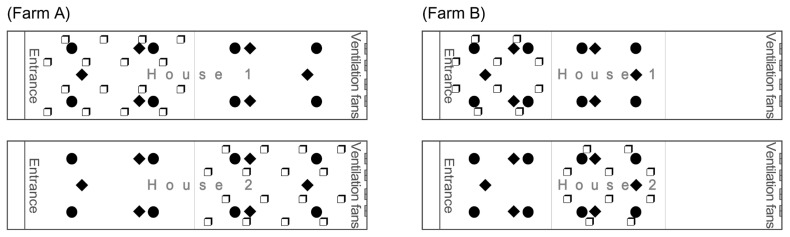
Schematic diagram of the sampling locations and placement of pecking blocks in houses. The symbol ‘❒’ represents the placement of enrichment (grain-based pecking block), while the symbols ‘◆’ and ‘⚫’ represent specific sampling locations. The ‘◆’ symbols indicate where productivity was measured, and the ‘⚫’ symbols indicate where feather and fecal corticosterone, footpad dermatitis, hock burn, feather dirtiness, litter, body surface temperature, and droppings for volatile fatty acids were measured.

Grain-based pecking blocks were manufactured to motivate pecking behaviors of broilers (Sinaebio Co., Ltd., Sungnam, Korea). The cube-shaped blocks sized 25 × 25 × 25 cm (Figure 1). The block consisted of 50% to 60% of by-products as brans, 10% to 20% of grains, 10% to 15% of limestone, and other ingredients (moisture, molasses, and glycerin). All ingredients were mixed before molding them in a cube-shaped mold. Pecking blocks were analyzed to contain moisture 13.71%±0.09%, crude protein 10.63%±0.08%, crude fat 3.53% ±0.12%, NDF 27.59%±0.08%, and ash content 13.94%±0.09%. One block per 1,000 birds was provided within the house [9]. In Farm A, the house had two equal regions with raised plastic net. Because half of the house had reared 16,000 birds, 16 pecking blocks were provided, while the other half was not provided with pecking blocks. In Farm B, the house had three equal regions with raised plastic net. Likewise, 10 blocks per one thirds of house were placed, while the other two regions were not provided with pecking blocks. Location of pecking blocks was alternated in different regions per house in both farms (Figure 2).

### Farm visit

On-farm welfare evaluation was assessed at 13 and 26 days of age for each farm. At 13 days of age, litter quality was analyzed and at 26 days of age, productivity, and physiological and welfare indicators were evaluated. Farm A had hatched chicks on May 7, 2022, and visits on May 19 and June 1. Farm B had hatched chicks on May 9, and visits on May 21 and June 3. During each visit, the indoor observations were performed.

### Measurements and assessments

#### Productivity

Body weight and flock uniformity at 26 days were investigated. One hundred birds were randomly selected and measured at three locations with or without pecking blocks per house to calculate for uniformity (expressed as coefficient of variation) using the mean values by location.

#### Corticosterone in feathers and fecal droppings

Corticosterone concentrations were analyzed in freshly voided fecal droppings and feathers. At the age of 26 days, feathers from 5 birds per location were sampled from 4 locations per treatment per house (8 samples per treatment per farm) and 0.05 g of feathers were collected from the interscapular area. The collected feathers were pretreated using the methods of Bortolotti et al [10] and Ataallahi et al [11] as a methanol-based extraction method, and were stored at −20°C until analysis. At 26 days of age, freshly voided fecal droppings were sampled at 8 locations per farm per treatment. The collected feces were pre-treated using the method of Sundbom et al [12] with an ethanol-based extraction method, and stored at −20°C until analysis.

Corticosterone in the extracted samples was analyzed using the commercial corticosterone ELISA kit (Catalog no. ADI-901-097; Enzo Life Sciences, Farmingdale, NY, USA). The absorbance was measured at 405 nm using a spectrophotometer (Synergy 2; BioTek Instruments, Inc., Winooski, VT, USA).

#### Footpad dermatitis, hock burn, and feather dirtiness score

Footpad dermatitis, hock burn, and feather dirtiness were analyzed per the Welfare Quality Assessment protocol for poultry (2017). At 26 days of age, 80 birds from 8 locations in each treatment (10 bird/location × 4 locations × 2 houses) were photographed in the order of footpad, hocks, and overall feathers with a digital camera. Footpad dermatitis and hock burn were evaluated on a 3-point scale in this study (score 0 to 2). Score 0 indicated no abnormality, score 1 indicated a minimal symptom, and score 2 indicated a noticeable symptom. Feather dirtiness was evaluated on a 3-point scale (score 0 to 2), where score 0 indicated clean, score 1 indicated slightly dirty, and score 2 indicated conspicuously dirty.

#### Gait score

As another animal welfare indicator, the gait score was evaluated on a 3-point scale according to the AssureWel Meat chicken assessment protocol (2016) in consideration of the effectiveness and practicality of evaluation on the farm. Broilers with abnormal gait were observed while walking 20% of the area within the house, and the flocks were evaluated after taking a video with a digital camera along the movement route. As for the assessment score, score 3 was classified as having abnormal gait and uncomfortable movement ability, score 4 was classified as severe gait abnormality and only a few steps were possible, and score 5 was classified as impossible to walk.

#### Litter moisture, nitrogen, and pH

The litter (ca. 100 g per location) was sampled from 8 locations per treatment per farm (1 sample/location × 4 locations × 2 houses). The sampled samples were stored at −20°C in a sealed plastic bag. Moisture in the litter was weighed in 50 g each of aluminum plates, dried in a dry oven at 135°C for 2 hours, and evaporated moisture was immediately measured. The nitrogen in the litter was analyzed by the Kjeldahl method on a sample dried at 60°C for 3 days in a dry oven. The pH of the litter was analyzed with a pH meter (Lab 845; SI analytics, Mainz, Germany) by mixing 2 g of a litter sample with 40 mL of distilled water referring to the method of Coufal et al [13].

#### Body surface temperature

Body surface temperature was taken with a thermographic camera (E8-XT; Teledyne FLIR, Wilsonville, OR, USA) from a distance of about 0.5 to 1.0 m to broilers. For 26-day-old broilers, 80 birds at 8 locations in each treatment were photographed (10 birds/location × 4 locations × 2 houses). The emissivity was 0.95 and the reflected temperature was 20°C during the shooting, and then the temperature of the head, chest, and legs of the photographed image was recorded using the software (FLIR Tools, ver. 6.4) provided by the thermographic camera manufacturer.

#### Volatile fatty acids in fecal samples

Fecal samples used for fecal corticosterone were used to measure volatile fatty acids (VFAs) as described by the method of Kim et al [14]. In brief, 1 g of collected excrement was homogenized in 4 mL of cold sterile phosphate-buffered saline (PBS), and 0.05 mL of saturated HgCl_2_, 1 mL of 25% H_3_PO_4_, and 0.2 mL of 2% pivalic acid were added to the homogeneous solution and centrifuged at 4°C for 20 minutes. One milliliter of supernatant was analyzed by gas chromatography (6890 Series GC System; HP, Palo Alto, CA, USA) to measure the concentration of VFAs, and quantitative analysis and relative ratio were calculated.

### Statistical analysis

Sampling locations per treatment were considered experimental replicate. Data were analyzed using the SAS (SAS Inst. Inc., Cary, NC, USA). Statistical analysis of the data was compared using the student *t*-test and was tested at the 5% significance level. A chi-square test was performed for the data on animal welfare indicators and Fisher’s Exact Test was performed when the expected frequency was less than 5 in the chi-square test.

## RESULTS AND DISCUSSION

### Body weight and uniformity

Table 2 shows the results of the effect of grain-based pecking block on body weight and flock uniformity at 26 days of age. During the experiment, all pecking blocks provided were consumed. It is calculated that chickens consumed approximately 9.2 g per bird during 26 days. As a result, no significant effect by pecking block on body weight and flock uniformity was noted both in Farms A and B.

Several studies reported that enrichments such as perches, barriers or hay bales had no effect on growth performance [15,16], It was also reported that providing grains on litter did not affect productivity [17]. This study is in line with other studies showing the lack of effect by pecking blocks in broiler chickens. It should be kept in mind that both farms had same breed, starter and finisher diets, perches, and wider stocking densities which may restrict additional benefit by pecking blocks, if any, on productivity.

### Concentrations of corticosterone in fecal droppings and feathers

Table 3 shows the results of the effect of pecking blocks on fecal and feather corticosterone in broilers at 26 days of age. No significant effect of pecking blocks on concentrations of corticosterone in feathers and fecal droppings was noted. Corticosterone concentrations ranged from 21.9 to 27.3 pg/mg in fecal dropping and from 5.92 to 8.11 pg/mg in feathers. Corticosterone is known as an indicator of biological stress that can be identified in various species including poultry [10,18]. Fecal droppings and feathers are often used to measure corticosterone in a non-invasive way [8,19]. In line with our study, Biasato et al [20] reported that there was no difference in fecal corticosterone between enrichment and control groups when insect larvae was used as enrichment for broilers. Meyer [21] also reported that there was no effect of additional environments such as laser light and platform on broiler plasma corticosterone. Although corticosterone is well known as stress hormone, corticosterone *per se* could not be considered the best indicator of welfare status [18]. Further studies might be needed to elucidate enrichment-mediated, if present, stress responses in stress environments such as stocking density or adverse ambient environments (cold or heat stress).

### Footpad dermatitis, hock burn, and feather dirtiness

**Figure 3:**
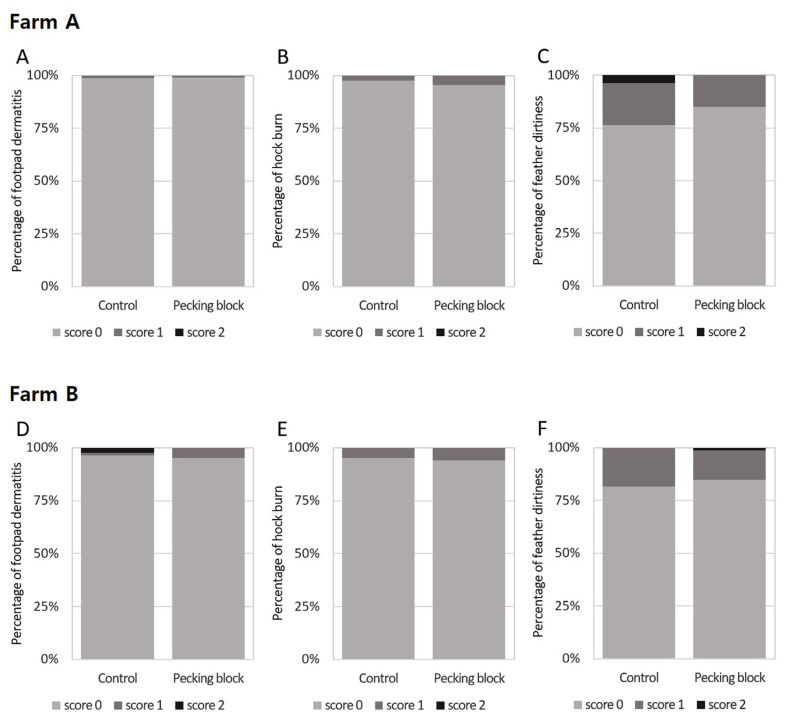
Distribution of broiler assessment results according to the level of footpad dermatitis (A, D), hock burn (B, E), and feather dirtiness (C, F) between the control and enrichment (grain block) groups at 26 days of age on Farm A (A-C) and Farm B (D-F). Footpad dermatitis (*χ*^2^ = 0.003), hock burn (*χ*^2^ = 0.552), and feather dirtiness (*χ*^2^ = 4.160) were measured in an average of 81 birds/house in the control group and 87 birds/house in the enrichment group on Farm A, according to the Welfare Quality Assessment protocol for poultry (2017). Footpad dermatitis (*χ*^2^ = 3.762), hock burn (*χ*^2^ = 0.072), and feather dirtiness (*χ*^2^ = 1.499) were measured in an average of 81 birds/house in the control group and 85 birds/house in the enrichment group on Farm B. Higher scores indicate negative outcomes for welfare and health. Fisher’s exact test was used instead of the *χ*^2^-test if at least one expected frequency was less than 5 between the treatments. No significant differences were found in the assessments.

Figure 3 shows the results of assessing the effect of pecking blocks on footpad dermatitis, hock burn, and feather dirtiness score for broilers at 26 days of age. It was evaluated according to the Welfare Quality Assessment protocol for poultry (2009), and the feather score evaluated the dirtiness of feathers. Results indicated that none of parameters (i.e., footpad dermatitis, hock burn, and feather dirtiness) was affected by pecking blocks both in Farms A and B.

Factors contributing to footpad dermatitis and hock burn include broiler weight and litter, being the latter caused by high humidity and ammonia from feces [22,23]. Little is known about the effects of pecking blocks on contact dermatitis in broilers. However, enrichments such as perch and hay bale in male chickens did not affect footpad dermatitis compared with those without enrichment [24], albeit that they relieved footpad dermatitis of female broilers indicating the gender-specific effect by enrichments. Tahamtani et al [23] reported that feeding corn roughage and dry bale tended to increase plantar dermatitis in 35-day-old broilers, although hock burns were not affected by enrichment provision. In addition, it has been reported that enrichment such as perches or platforms could reduce footpad dermatitis [23,25]. Differences between our and earlier studies might be due to breed, gender, stocking density, and age which our study used wide stocking density and measured the indicators at 26 day-day-old broilers.

### Gait score

**Figure 4:**
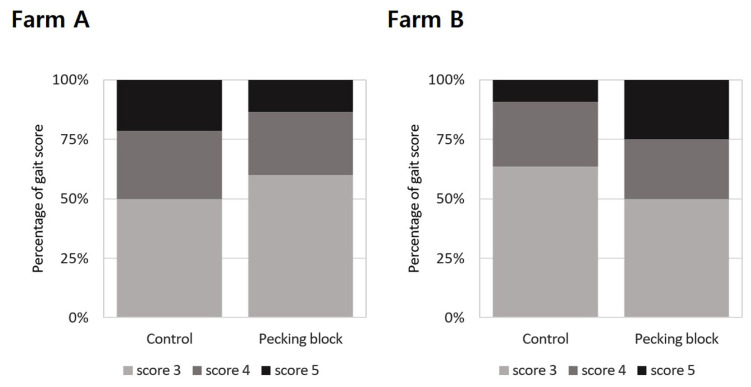
Distribution of broiler assessment results according to the level of gait score between the control and enrichment (grain block) groups at 26 days of age on Farm A and B. Gait scores (Farm A, *χ*^2^ = 0.450; Farm B, *χ*^2^ = 1.353) were assessed in 20% of the flock per house using the AssureWel Meat Chicken Assessment Protocol (2016). Higher scores indicate poorer welfare and health outcomes. Fisher’s exact test was used rather than the *χ*^2^-test if at least one expected frequency between the treatments was less than 5. No significant differences were found in the assessment.

Figure 4 shows the results of assessing the effect of pecking blocks on gait score for broilers at 26 days of age. Result showed that there was no significant difference in gait score between treatment groups in Farms A and B. In Farm B, the gait scores seemed to be higher in the pecking block-provided broilers compared with the control group. However, it should be emphasized that broilers exhibited over the scores of 3, 4, and 5 were recorded to be 4, 2, and 2 for the pecking block group while 7, 3, and 1 for the control group, leading to insignificant differences between the treated groups.

The gait score of broilers is an animal welfare assessment indicator based on leg weakness or lameness [6]. Experiment conducted by Bailie and O’Connell [26] showed that perches and hanging straps as pecking objects were effective in behavioral and welfare aspects although they did not appear to affect leg health. Thus, it is likely that pecking objects have more impact on various behavior such as pecking and walking, rather than leg health. Unfortunately, monitoring the behavior of broilers with pecking blocks was not attempted in this experiment. It is however clearly seen that pecking blocks accelerated pecking behavior by consuming them. Chickens are estimated to have ingested 9.2 g per bird during 26 the days, as described previously. In addition, it should be kept in mind that gait scores were kept low in all treatments of Farms A and B. This might be related to body weights at the time of welfare evaluation that had been tested at 26 days of age.

### Litter quality

Table 4 shows the results of the effect of pecking blocks on the moisture, nitrogen and pH of the litter samples. Results indicated that there was no significant difference by pecking blocks provision in litter quality between treatment groups in Farms A and B.

Broiler litter is a mixture of manure, bedding material, wasted feed, feathers, and soil. Litter moisture is the parameters to assess animal welfare as it affects footpad dermatitis and hock burn [27]. Nitrogen in the litter increased with ages, which is then released as ammonia through a series of mechanisms [28]. The study suggests that the provision of pecking blocks has no effect on litter nitrogen and pH. De Jong and Gunnink [29] reported that there was no difference in litter quality between the control and enrichment treatment groups that provided with sawdust bales, perches, and metal chains as pecking objects. Spieß et al [30] found that enrichment consisting of perches and platforms had no significant effect on litter moisture. Mocz et al [31] also reported that the enrichment environment consisting of platforms and hay bales did not affect litter moisture. These studies, combined with our findings, suggest that enrichment provision has little, if existed, impact on litter quality.

### Body surface temperature

Table 5 shows the results of the effect of pecking blocks on body surface temperature for broilers at 26 days of age. The temperature humidity index (THI) according to the temperature and humidity within the house at the time of measurement was not different and ranged from 80.3 to 80.9 in Farm A and from 89.9 to 90.2 in Farm B. Of interest, the body surface temperature of head (p = 0.029) and legs (p = 0.011) in the enrichment vs. control groups was significantly higher in Farm A. However, enrichment-mediated increase in body surface temperature seen in Farm A was not found in Farm B.

The body surface temperature of broilers indicates the state of body temperature regulation for external environmental conditions, and it is known that the body surface temperature of the head (especially around the eyes) has a high positive correlation with body core temperature [32,33]. Yildirim and Taskin [34] reported that rectal temperature measured at 21 and 42 days was not altered in broiler chickens provided with perch, a ball, and a mirror as environmental enrichment. Thus, it is difficult to conclude that the pecking blocks *per se* increased body surface temperature of broiler chickens. At this stage, no clear explanation on elevated body surface temperature in pecking block-provided chickens is readily available.

### Volatile fatty acids in feces

Table 6 shows the results of the effect of pecking blocks on fecal VFAs for broilers at 26 days of age. As pecking blocks contains grain ingredients, fecal VFAs were analyzed on the hypothesis that ingestion of these grain-based pecking blocks would affect the intestinal microbial communities. It might be understood that pecking blocks used in this study might be one of many factors affecting the concentrations of VFAs. In contrast to our expectation, the absolute concentration of VFAs in Farms A and B was not altered whether pecking blocks were provided or not. However, in Farm B, the relative butyrate concentration was elevated in broilers provided with pecking blocks compared with those provided with no pecking blocks (p = 0.023).

VFAs are metabolic products produced by bacterial fermentation on dietary fiber and undigested feed- and host-origin proteins [35]. VFAs are mainly acetate, propionate and butyrate, and many studies have found that VFAs play an important role in regulating the intestinal health of poultry [36,37]. It is proposed that the status of intestinal health in chickens can be determined by analyzing VFAs produced by anaerobic bacteria in the gut [37]. Various studies have reported that butyrate improved disease resistance by inhibiting the growth of intestinal bacteria such as Salmonella and Clostridium [38], and has a positive effect on the overall health of broilers by affecting the intestinal microbial composition and intestinal mucosal function [39]. Based on the findings, it is expected that grain-based pecking blocks can be used to improve gut health of chickens raised in welfare broiler farms. This is the first report to see the pecking blocks-mediated increase in butyrate concentration in fecal droppings. It is however pointed out that pecking blocks did not equally influence the indicators of gut health in both farms. Clear explanation on the inconsistent results seen by the pecking blocks in both farms is not readily available. Further studies are warranted whether grain-based pecking blocks would modulate gut health, immunity, and microbiome in broiler chickens raised in different environments such as used vs fresh litter.

## CONCLUSION

The aim of this study was to investigate the effect of grain-based pecking blocks as enrichment on productivity and animal welfare status of commercial broiler chickens. Providing pecking blocks did not affect productivity (e.g., body weight and uniformity) and animal welfare indices (e.g., footpad dermatitis, hock burn, feather dirtiness, gait score, and litter quality). On the other hand, pecking blocks increased body surface temperature and relative percentages of butyrate in fecal droppings compared with those provided with no pecking blocks. It is concluded that grain-based pecking blocks did not affect welfare indicators, but might improve gut health of broiler chickens.

## Figures and Tables

**Table 1:** Characteristics of commercial experimental farms

Items	Farms	

	Farm A	Farm B
Farm location	Boseong-gun, Jeollanam-do, South Korea	Yeongam-gun, Jeollanam-do, South Korea
Strain	Arbor Acres	Arbor Acres
House type	Windowless	Windowless
Ventilation type	Forced exhaust	Forced exhaust
Flock size, number of birds	32,000	26,800
House size (m, m^2^)	120×16 = 1,920	100×16 = 1,600
Stocking density (birds/m^2^)	16.7	16.8
Litter type and recycling	Fresh rice hulls	Recycled rice hulls
Perches installed	Wood, 2 m per 1,000 birds	Wood, 2 m per 1,000 birds
Photoperiod^1)^	Gradually from 23L:1D to 18L:6D in the first 5 days, and continued at 18L:6D	Gradually from 23L:1D to 18L:6D in the first 5 days, and continued at 18L:6D

1) The photoperiod is represented as the number of hours of light (L):darkness (D).

**Table 2:** Body weight and uniformity of 26-day-old broilers with or without enrichment^1)^

Items	Treatment				p-value

	Control		Enrichment		
	
	Mean	SD^2)^	Mean	SD^2)^	
Farm A					
Body weight (g/bird)	1,224	43.2	1,218	49.0	0.850
CV^3)^ (%)	4.09	0.45	3.66	1.08	0.389
Farm B					
Body weight (g/bird)	1,298	30.0	1,297	34.0	0.960
CV^3)^ (%)	4.60	0.89	4.15	1.49	0.575

1) Values are means of 6 replicates per treatment.

2) SD, standard deviation.

3) CV, coefficient of variation (standard deviation/average weight of 100 broilers at 26 days of age per 1 location).

**Table 3:** Corticosterone concentrations in broiler feces and feathers with or without enrichment^1)^

Items	Treatment				p-value

	Control		Enrichment		
	
	Mean	SD^2)^	Mean	SD^2)^	
Farm A					
Feces (pg/mg)	21.85	6.84	27.25	2.25	0.115
Feather (pg/mg)	6.45	0.93	5.92	0.71	0.226
Farm B					
Feces (pg/mg)	23.73	2.16	24.80	5.59	0.668
Feather (pg/mg)	7.01	1.50	8.11	1.35	0.142

1) Values are means of 8 replicates per treatment.

2) SD, standard deviation.

**Table 4:** Litter moisture, nitrogen, and pH in broiler farmhouses with or without enrichment^1)^

Items	Treatment					p-value

	Control			Enrichment		
	
	Mean	SD^2)^		Mean	SD^2)^	
13 days						
Farm A						
Moisture (%)	28.9	2.82		26.5	4.74	0.247
Nitrogen (%)^3)^	1.56	0.19		1.51	0.15	0.574
pH	7.50	0.22		7.42	0.23	0.512
Farm B						
Moisture (%)	21.9	1.62		21.1	1.60	0.323
Nitrogen (%)^3)^	2.38	0.15		2.51	0.15	0.106
pH	8.45	0.20		8.36	0.25	0.311
26 days						
Farm A						
Moisture (%)	23.6	1.87		25.1	2.30	0.177
Nitrogen (%)^3)^	2.10	0.09		2.13	0.19	0.741
pH	6.86	0.11		6.81	0.23	0.595
Farm B						
Moisture (%)	26.4	1.06		25.7	1.63	0.288
Nitrogen (%)^3)^	2.69	0.07		2.71	0.08	0.636
pH	6.02	0.24		6.20	0.14	0.107

1) Values are means of 8 replicates per treatment.

2) SD, standard deviation.

3) On an as-is basis.

**Table 5:** Body surface temperature in broiler chickens with or without enrichment^1)^

Items	Treatment^2)^				p-value

	Control		Enrichment		
	
	Mean	SD^3)^	Mean	SD^3)^	
Farm A (°C)					
Head	38.5b	0.35	39.0a	0.46	0.029
Chest	31.7	0.55	32.1	0.47	0.154
Legs	38.0^b^	0.80	39.0^a^	0.49	0.011
Farm B (°C)					
Head	38.9	0.38	39.0	0.53	0.603
Chest	32.9	0.64	32.9	0.75	0.834
Legs	39.7	0.26	39.7	0.57	0.927

1) Values are means of 8 replicates.

2) Farm A (control) = temperature 28.9°C, humidity 29.2%, THI 80.9; Farm A (enrichment) = temperature 28.5°C, humidity 29.8%, THI 80.3; Farm B (control) = temperature 33.9°C, humidity 40.3%, THI 90.2; Farm B (enrichment) = temperature 33.7°C, humidity 41.2%, THI 89.9.

3) SD, standard deviation.

a,b Means with a different superscript differ (p<0.05).

**Table 6:** Fecal volatile fatty acids in broiler chickens with or without enrichment^1)^

Items	Treatment				p-value

	Control		Enrichment		
	
	Mean	SD^2)^	Mean	SD^2)^	
Farm A					
mmol/kg feces					
Acetate	19.78	7.27	18.09	3.27	0.557
Propionate	3.20	0.59	3.14	1.73	0.928
Isobutyrate	1.11	0.63	1.16	0.60	0.921
Butyrate	2.76	1.32	2.30	1.09	0.459
Isovalerate	1.60	0.92	1.76	1.12	0.778
Valerate	2.09	0.59	1.95	0.95	0.780
SCFA^3)^	25.74	8.41	23.52	4.75	0.527
Total VFA^4)^	31.19	8.95	28.18	5.56	0.465
% of total VFA					
Acetate	70.59	8.86	71.56	11.83	0.856
Propionate	13.73	3.61	12.71	5.31	0.662
Isobutyrate	3.02	2.15	3.42	0.87	0.775
Butyrate	9.74	3.43	8.51	2.65	0.438
Isovalerate	5.92	2.33	6.22	3.31	0.844
Valerate	5.66	1.65	7.26	2.90	0.304
SCFA^3)^	94.06	7.84	92.78	8.87	0.765
Farm B					
mmol/kg feces					
Acetate	22.60	12.35	24.30	11.01	0.776
Propionate	3.65	1.33	4.38	1.67	0.352
Isobutyrate	1.86	0.96	1.10	0.24	0.251
Butyrate	1.78	0.96	2.78	1.36	0.108
Isovalerate	1.33	0.66	2.01	1.21	0.180
Valerate	1.63	1.21	1.48	0.71	0.777
SCFA^3)^	28.02	14.44	31.46	13.45	0.630
Total VFA^4)^	33.61	16.20	35.36	15.41	0.834
% of total VFA					
Acetate	72.35	4.34	67.89	5.42	0.091
Propionate	14.34	3.83	14.07	3.17	0.878
Isobutyrate	2.59	0.63	3.54	1.40	0.452
Butyrate	5.85^b^	0.53	8.37^a^	2.46	0.023
Isovalerate	5.17	3.06	5.62	1.66	0.720
Valerate	4.68	1.75	4.82	2.23	0.896
SCFA^3)^	92.54	3.55	90.33	3.71	0.244

1) Values are means of 8 replicates per treatment.

2) Standard error of the means.

3) Short-chain fatty acids (acetate+propionate+butyrate).

4) Total volatile fatty acids (acetate+propionate+butyrate+isobutyrate+valerate+isovalerate).

a,b Means with a different superscript differ (p<0.05).
